# ChIP-Seq analysis of the adult male mouse brain after developmental exposure to arsenic

**DOI:** 10.1016/j.dib.2015.08.037

**Published:** 2015-09-09

**Authors:** Christina R. Tyler, Jessica A. Weber, Matthew Labrecque, Justin M. Hessinger, Jeremy S. Edwards, Andrea M. Allan

**Affiliations:** a Department of Neurosciences, University of New Mexico Health Sciences Center, Albuquerque, NM 87131, USA; b Department of Biology, University of New Mexico, Albuquerque, NM 87131, USA; c Department of Molecular Genetics and Microbiology, University of New Mexico, Albuquerque, NM 87131, USA; d Department of Chemical & Nuclear Engineering, University of New Mexico, Albuquerque, NM 87131, USA; e Department of Chemistry & Chemical Biology, University of New Mexico, Albuquerque, NM 87131, USA; f Cancer Research & Treatment Center, University of New Mexico Health Sciences Center, Albuquerque, NM 87131, USA

## Abstract

Exposure to the common environmental contaminant arsenic impacts the epigenetic landscape, including DNA methylation and histone modifications, of several cell types. Developmental arsenic exposure (DAE) increases acetylation and methylation of histone proteins and the protein expression of several chromatin-modifying enzymes in the dentate gyrus (DG) subregion of the adult male mouse brain [26]. To complement and support these data, ChIP-Seq analysis of DNA associated with trimethylation of histone 3 lysine 4 (H3K4me3) derived from the adult male DG after DAE was performed. DAE induced differential H3K4me3 enrichment on genes in pathways associated with cellular development and growth, cell death and survival, and neurological disorders, particularly as they relate to cancer, in the adult male brain. Comparison of H3K4me3 enrichment in controls revealed mechanisms that are potentially lacking in arsenic-exposed animals, including neurotransmission, neuronal growth and development, hormonal regulation, protein synthesis, and cellular homeostasis. New pathways impacted by arsenic include cytoskeleton organization, cell signaling, and potential disruption of immune function and warrant further investigation using this DAE paradigm in the mouse brain.

**Specifications Table**

**Table:** 

Subject area	*Biology*
More specific subject area	*Neurotoxicology and epigenetics*
Type of data	*Tables, graph*
How data was acquired	*Genomic sequencing (HiSeq2000) and bioinformatics*
Data format	*Filtered for peak annotation, and analyzed for differential enrichment and gene ontology*
Experimental factors	*Developmental exposure to 50 parts-per-billion sodium arsenate in drinking water (DAE)*
Experimental features	*Exposure to sodium arsenate (50 parts-per-billion, ppb) in drinking water occurred during all three trimesters of fetal/neonatal development in C57BL/6 mice. Arsenic exposure ceased after the third postnatal trimester of development at approximately postnatal day 23. Animals were then placed on tap water (consisting of 5 ppb arsenic) until adulthood. Brain tissue, specifically the dentate gyrus sub-region of the hippocampus, was harvested from control and arsenic-exposed adult male mice (postnatal day 70). Chromatin immunoprecipitation was performed for the histone 3 lysine 4 trimethylation modification; immunoprecipitated DNA was isolated and sent to the University of Southern California for sequencing analysis on a HiSeq2000. Data was analyzed for specific H3K4me3 enrichment after arsenic exposure compared to controls.*
Data source location	*N/A*
Data accessibility	*Filtered raw data files from sequencing provided in table format Analyzed data files provided in table format Analyzed data files provided in graphical format*

## Background

The data provided in this article are intended to support research demonstrating that developmental arsenic exposure (DAE) increases the levels of H3K4me3 and H3K9ac histone modifications along with associated histone methyltransferase and acetyltransferase proteins in the dentate gyrus (DG) of the adult mouse brain [26]. The DG contains neural progenitor cells that actively undergo proliferation, differentiation and integration into the hippocampal neural circuitry in adulthood [19]. These processes are collectively referred to as adult neurogenesis and are important for cognitive function and disease susceptibility including depression [15]. Epidemiological studies have shown arsenic exposure, a common contaminant found in drinking water, correlates with cognitive dysfunction, particularly in children, and psychiatric disorders like depression in adults [3,30,4,5]. Our DAE paradigm reduces differentiation of neural progenitor cells, induces deficits in memory, and increased depressive behaviors in adult male mice [18,24,27]. Epigenetic mechanisms within neural progenitor cells, particularly histone modifications, are paramount for proper specification of gene expression for all the processes of neurogenesis [11,14]. Arsenic exposure has been shown to alter histone modifications in the blood of humans exposed to high levels of this toxin [22,29,7]. Thus, to determine potential mechanisms of arsenic-induced toxicity in the DG, chromatin immunoprecipitation followed by sequencing (ChIP-Seq) for histone 3 lysine 4 trimethylation (H3K4me3) was performed. While research demonstrating arsenic’s impact on the brain’s epigenome brain is limited to a handful of studies [10,17,28], several reports have established that arsenic adversely alters histone posttranslational modifications and DNA methylation in the mammalian body [13,17,22,29,7–9]. While arsenic speciation likely plays a role in damage to methylation capacity in the body [16], generally, excessive exposure to arsenic inhibits one-carbon metabolism, effectively depleting S-adenosyl methionine (SAM) [21]. However, both hypo- and hyper-methylation of DNA has been observed in response to arsenic toxicity [20]; as such, simple depletion of SAM and altered methylation status is likely not the mechanism of arsenic toxicity in context of the brain [23]. Using ChIP-seq analysis we sought to identify new pathways for mechanisms of action, particularly in this region of the brain that contains stem cells. As arsenic exposure has been shown to adversely impact males more than females, this analysis was performed on the male brain [25].

## Value of the data

• First H3K4me3 ChIP-seq analysis in the dentate gyrus of a mouse model.
• First genomic data to demonstrate that developmental arsenic exposure induces long-lasting transcriptional activation via altered epigenetic status in the adult male mouse brain.
• Analyses indicate that arsenic alters epigenetic regulation of genes involved in cell death and survival, cell development and growth, abnormal cell morphology and organization, gene expression, some immune function, and a host of neurological diseases, including cancer and neuropathy, in the brain.

## Data

Using a mouse model of developmental arsenic exposure (DAE), we have previously shown deficits in learning and memory, depressive-like symptoms, and reduced adult neurogenesis in adult male mice [18,24,27]. A region important in these processes is the dentate gyrus (DG) of the hippocampus, for which we have shown that arsenic increases H3K4 trimethylation and alters protein expression of MLL and KDM5B, two H3K4me3 chromatin modifiers [26]. To complement this data, next generation sequencing of H3K4me3 enriched DNA from the DG of control and arsenic-exposed male mice was performed on an Illumina HiSeq 2000 with 50 bp single end reads with a 98% alignment of approximately 30 million reads to the mouse genome. Peak calling comparisons between the arsenic and control sequences were performed using the HOMER (Hypergeometric Optimization of Motif EnRichment) package; annotation of peaks differentially enriched for H3K4me3 in the arsenic sequences and in the control sequences, along with the most significant gene ontology (GO) categories and functional annotations, are provided.

**Fig. 1:**
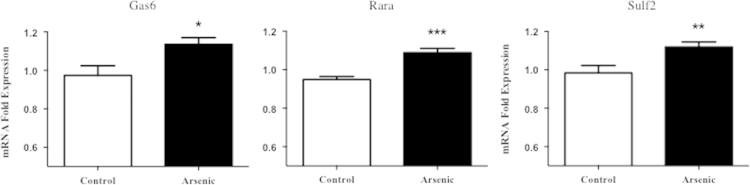
mRNA gene expression validation of ChIP-Seq analysis in the adult male dentate gyrus after developmental arsenic exposure.

The data is as follows:

1. Pdf files of the original sequencing reads that have been filtered, aligned, and annotated to the mouse genome (mm10), indicating all genes with H3K4me3 enrichment in both the control and arsenic-exposed animals relative to input, labeled as the following
  a. Supplementary Table S1: H3K4me3 ChIP-Seq for arsenic-exposed adult male dentate gyrus (PD70), Arsenic sample 1
  b. Supplementary Table S2: H3K4me3 ChIP-Seq for arsenic-exposed adult male dentate gyrus (PD70), Arsenic sample 2
  c. Supplementary Table S3: H3K4me3 ChIP-Seq for control adult male dentate gyrus (PD70), Control sample 1
  d. Supplementary Table S4: H3K4me3 ChIP-Seq for control adult male dentate gyrus (PD70), Control sample 2
2. Pdf files comparing the H3K4me3 enrichment of arsenic versus control animals and the significant gene ontologies, labeled as the following:
  a. Supplementary Table S5: HOMER Analysis of H3K4me3 differential peak binding after developmental arsenic exposure in adult male dentate gyrus samples
  b. Supplementary Table S6: Significant Gene Ontology categories and molecular functions annotations for genes with differential H3K4me3 peak enrichment in the adult male dentate gyrus after developmental arsenic exposure from HOMER assessment in Supplementary Table S5
3. Pdf files comparing the H3K4me3 enrichment of control versus arsenic animals and the significant gene ontologies, labeled as the following:
  a. Supplementary Table S7: HOMER Analysis of H3K4me3 differential peak binding in control male dentate gyrus samples
  b. Supplementary Table S8: Significant Gene Ontology categories and molecular functions annotations for genes with differential H3K4me3 peak enrichment in the adult male dentate gyrus of unexposed animals (control) from HOMER assessment in Supplementary Table S7
4. Graphs of gene expression validation for Gas6, Rora, Sulf2 genes labeled as:
  a. Fig. 1. mRNA gene expression validation of ChIP-Seq analysis in the adult male dentate gyrus after developmental arsenic exposure
  i. ChIP-Seq analysis indicated these genes have increased H3K4me3 enrichment in arsenic-exposed animals; mRNA expression of these three genes is significantly increased in the adult male dentate gyrus after developmental arsenic exposure validating results provided by sequencing analysis

## Experimental design

A mouse model of developmental arsenic exposure during all three-trimester equivalents was used, as previously described [18,24], to determine the impact of arsenic on the epigenome. DNA from H3K4me3 chromatin immunoprecipitation of the dentate gyrus region of the male brain was sequenced using via HiSeq2000 and analyzed using HOMER software suite and Ingenuity Pathway Analysis. A selection of genes with significant H3K4me3 enrichment in either control or arsenic-exposed animals was analyzed for mRNA expression in dentate gyrus tissue to confirm ChIP-Seq findings.

### Developmental arsenic exposure paradigm

The Institutional Animal Care and Use Committee at the University of New Mexico (UNM) approved the animal protocols, including the arsenic exposure paradigm, used in this study. C57BL/6 mice obtained from Jackson Labs were maintained on a reverse light/dark cycle (lights off at 0800) with *ad libitum* access to food and water in the Animal Resource Facility at UNM. Arsenic exposure was performed as previously described [27]; briefly, singly-housed female mice aged 55 days were acclimated to drinking 50 parts-per-billion arsenic water (sodium arsenate, Sigma Aldrich) for 10 days prior to mating. Arsenic water was prepared weekly using standard tap and MilliQ water. Control mice were administered tap water from UNM, which contains approximately 2–5 ppb arsenic. Mating occurred for five days; dams continued to drink arsenic-laced water throughout pregnancy until offspring were weaned at postnatal day (PD) 23. Offspring were group housed separately by sex, four per cage, with *ad libitum* access to food and tap water. At PD70, animals were euthanized via rapid decapitation, and the dentate gyrus from male animals was microdissected and snap frozen and stored at −80 **°**C until further analysis. For each experiment, *n* represents the number of different litters used with one animal per litter to avoid litter effects.

### Chromatin immunoprecipitation

Dentate gyrus tissue obtained from adult male mice was homogenized in a Biomasher II disposable microhomogenizer (Kimble Chase) using a 1% formaldehyde crosslinking solution (1% formaldehyde, 1 mM EDTA, 0.5 mM EGTA, 50 mM HEPES, pH 8.0) for 15 min. Reagents from the ChIP-IT^®^ Express Chromatin Immunoprecipitation Kits from Active Motif (53008) were used for some steps in the protocol. A 1× Glycine solution (Active Motif) was added to each reaction for 5 min. Homogenates were centrifuged at 1000×*g* for 6 min at 4 °C and washed with 1× PBS containing 1 μg/μl protease inhibitor cocktail (Sigma, P8340). Cell pellets were resuspended in cell lysis buffer containing 0.5% Triton-X 100 (v/v), 85 mM KCl, 5 mM HEPES, and 2 μg/μl protease inhibitor cocktail and allowed to sit on ice for 30 min. Cells were centrifuged as before, resuspended in cell lysis buffer, and repelleted as before. The pellet was resuspended in nuclear lysis buffer containing 50 mM Tris, 10 mM EDTA, 1% SDS, and 2 μg/μl protease inhibitor cocktail. Crosslinked chromatin was sheared via sonication. Samples were sonicated for 10 s with 2 min on ice between intervals (10×) to yield approximately 300–500 base pair chromatin, determined by 1.5% (w/v) agarose gel separation. Samples were centrifuged at 14,000×*g* for 10 min at 4 °C. The supernatant was snap frozen and stored at –80 **°**C. The antibody of interest was incubated with magnetic beads for 4 hours at 4 **°**C prior to the IP. ChIP-ready chromatin was incubated overnight with magnetic beads (Active Motif) and the histone 3 lysine 4 trimethyl (H3K4me3) antibody (Millipore, 04-745). Antibody validation was performed using RNA Polymerase II (Qiagen, GAM-111), IgG (Qiagen, GAM-8208), and the H3K4me3 antibody for the genes *Gapdh* and *Myod1* as positive and negative controls, respectively. A small fraction of the chromatin was saved to derive the input DNA. The following day, samples were washed at 4 °C and the DNA eluted using a ChIP DNA Purification Kit (Active Motif; 58002).

### ChIP-Seq library preparation and sequencing

The University of Southern California Norris Cancer Center Next Gen Sequencing Core Facility (http://epigenome.usc.edu) performed library preparation and sequencing on H3K4me3 immunoprecipitated DNA derived from control and arsenic-exposed adult male dentate gyrus tissue. Library preparation was performed using a NEB Ultra DNA library kit for biological replicates of control and arsenic samples (50 ng), and a separate library was generated from input DNA (no IP) from pooled tissue. Libraries were quantitated and size distribution and purity determined using a Qubit fluorometer and an Agilent 2100 Bioanalyzer, respectively. Sequencing was performed on an Illumina HiSeq 2000 sequencer using v3 chemistry with 50 bp single-end reads. Approximately 30 million reads per sample (two arsenic biological replicates, two control biological replicates, one input control) were obtained.

### ChIP-Seq analyses and gene ontology

The USC Sequencing Core Facility performed quality control, using the CASAVA pipeline (version 1.8.4) and mapping to the USCS mouse genome (mm10), using BWA (version 0.6.2). ChIP-seq peaks were identified and analyzed using the HOMER software suite (http://biowhat.ucsd.edu/homer/). Tag directories were made for each of the five samples, and the findPeaks function in histone mode, with the FDR threshold 0.001, was used to filter approximately 16,000−18,000 peaks in each of the four ChIP-Seq samples against the input sample. The getDifferentialPeaks function was then used to identify differentially bound peaks with greater than 4-fold H3K4me3 enrichment, with a cumulative Poisson *p*-value less than 0.0001, in the arsenic samples compared to the control samples and vice versa. Annotation of the enriched peaks was performed using the HOMER annotatePeaks with the mm10 mouse genome as a reference and promoters defined as −1 kb to +1 kb relative to the position of the TSS. Functional clustering using Ingenuity Pathway Analysis (IPA) was performed to determine common processes affected by developmental arsenic exposure.

### Gene expression validation

Trimethylation of lysine 4 on histone 3 (H3K4me3), particularly in the promoters and coding regions of genes, is associated with transcriptional activation [1,2]. To confirm results provided by ChIP-Seq data analysis via HOMER, we assessed mRNA gene expression for candidate genes with significant H3K4me3 enrichment in either arsenic-exposed or control dentate gyrus tissue. Tissue was derived from adult male mice (aged 70 days) from different litters than those used for sequencing to avoid litter confounds. Total RNA was purified from whole mouse dentate gyrus using the Ambion mirVana™ miRNA Isolation Kit (Life Technologies; AM1560) following the manufacturer’s instructions. Total RNA was quantified with the Qubit® RNA BR Assay Kit (Life Technologies; Q10211). gDNA was digested with DNase I recombinant (Roche Life Science; 04716728001) and cDNA reverse transcription was conducted with the Transcriptor First Strand cDNA Synthesis Kit (Roche Life Science; 04897030001). cDNA was quantified by NanoDrop 1000 spectrophotometer (Life Technologies). Quantitative PCR was performed on a Roche LightCycler 96 with Roche FastStart Essential DNA Green Master (06924204001) under standard cycle conditions. Results were analyzed by the relative quantification method using *Hprt* as an endogenous control. mRNA target primer efficiencies were validated and matched to control primer efficiencies. Primer sequences were as follows:

**Table:** 

**Gene**	**Gene ID**	**Forward primer**	**Reverse primer**	**Source**
Hprt (set a)	15452	TGACACTGGCAAAACAATGCA	GGTCCTTTTCACCAGCAAGCT	[12,6]
Hprt (set b)	15452	AAGACTTGCTCGAGATGTCATGAA	ATCCAGCAGGTCAGCAAAGAA	[6]
Gas6	14456	TGCTGGCTTCCGAGTCTTC	CGGGGTCGTTCTCGAACAC	Primer Bank ID: 9506715a1
Rara	19401	TTCTTTCCCCCTATGCTGGGT	GGGAGGGCTGGGTACTATCTC	Primer Bank ID: 22800363a1
Sulf2	72043	CTGCCACTATGGCTGCTGTC	GTTGGGCCGGATGTTCCTG	Primer Bank ID: 26330131a1

## Footnotes

Appendix A Supplementary data associated with this article can be found in the online version at doi:10.1016/j.dib.2015.08.037.
